# Tuberculosis of the Adrenal Gland: A Case Report and Review of the Literature of Infections of the Adrenal Gland

**DOI:** 10.1155/2014/876037

**Published:** 2014-08-06

**Authors:** Jagriti Upadhyay, Praveen Sudhindra, George Abraham, Nitin Trivedi

**Affiliations:** Department of Internal Medicine, Saint Vincent Hospital, Worcester, MA 01760, USA

## Abstract

Infections of the adrenal glands remain an important cause of adrenal insufficiency, especially in the developing world. Indeed, when Thomas Addison first described the condition that now bears his name over 150 years ago, the vast majority of cases were attributable to tuberculosis. Here we describe a classic, but relatively uncommon, presentation in the United States of adrenal insufficiency followed by a review of the current literature pertaining to adrenal infections.

## 1. Introduction

A 46-year-old man presented to his physician with a 3-month history of generalized weakness and 15-pound unintentional weight loss. He also reported mild dyspnea on exertion and decreased appetite. His past medical history was significant for hypertriglyceridemia, primary hypothyroidism, and vitamin D deficiency. He had emigrated from the Philippines 6 years prior and had been working as a nurse at a skilled nursing facility. He had not left the country since his initial arrival. He denied sick contacts, specifically exposure to tuberculosis, smoking, alcohol consumption, or the use of illicit substances. A tuberculin skin test performed in 2007 resulted in induration (diameter unknown) and it was attributed to prior BCG vaccine. There was no evidence of pulmonary tuberculosis on a chest radiograph. Physical examination revealed abdominal distension and free fluid but was otherwise unremarkable. A diagnostic paracentesis revealed an exudative effusion with a positive Ziehl Neelsen stain for acid fast bacilli. The patient was started on treatment (Isoniazid, rifampicin, pyrazinamide, and ethambutol) for presumed extrapulmonary tuberculosis which was later confirmed by culture.

**Figure 1:**
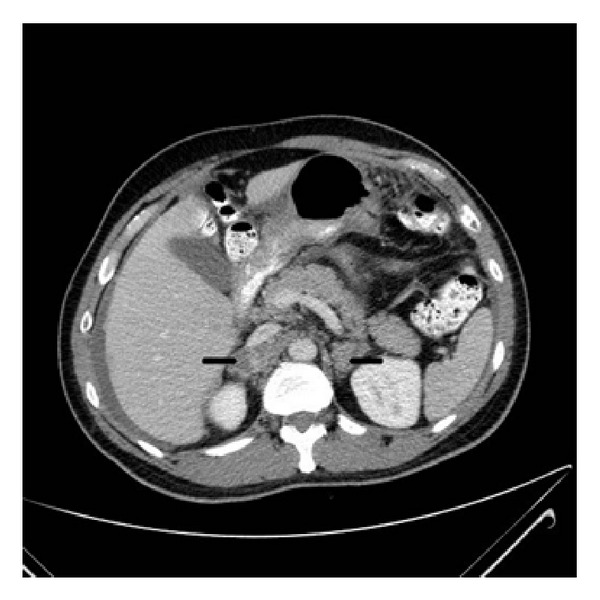
CT scan of the abdomen and pelvis with oral and intravenous contrast showing bilateral adrenal enlargement (black arrows).

One month after starting antitubercular therapy he presented to the hospital with worsening fatigue, salt craving, vomiting, loss of libido, and erectile dysfunction. On examination, he had low blood pressure and appeared cachectic. In addition he had bitemporal muscle wasting and hyperpigmentation of skin, oral mucosa, and nails. Laboratory evaluation was significant for hyponatremia, hyperkalemia, and mild hypercalcemia. A random cortisol was 2.5 mcg/dL with an ACTH of 531.2 pcg/mL. The basal and cosyntropin stimulated serum cortisol were, respectively 1.8 mcg/dL and 2.0 mcg/dL, which was consistent with the diagnosis of primary adrenal insufficiency most likely due to tuberculosis. A computed tomography scan of the abdomen with intravenous contrast revealed bilaterally enlarged adrenal glands (4 cm × 3.3 cm on the right, 2.3 cm × 2.1 cm on the left) (Figure 1). On review of his prior CT scan of the abdomen, the patient had bilaterally enlarged adrenal glands at the time of his initial presentation as well.

With the background of tuberculosis and acute adrenal insufficiency diagnosed by laboratory test, bilateral enlargement of adrenal glands was considered most consistent with tuberculosis in our patient. Deterioration of his clinical status following antitubercular treatment could be attributed to accelerated cortisol metabolism by induction of CYP 3A4 by rifampicin. He was initially treated with intravenous hydrocortisone and was subsequently discharged on hydrocortisone and fludrocortisone. His symptoms have improved significantly. However, he is requiring slightly higher dose of hydrocortisone, which could be due to CYP 3A4 induction by rifampicin. He is likely to require lifelong treatment for adrenal insufficiency. A study that looked at tuberculosis patients with bilaterally enlarged adrenal glands found that treatment with antituberculosis drugs does not improve or help recover adrenal functionality [1]. Adrenal biopsy was not performed because the presentation was strongly suggestive of adrenal tuberculosis with active extra-adrenal tuberculosis.

*Comment*. It is to be noted that BCG vaccine received at birth has no impact on PPD test result 10 years later [2]. The presumption made by the other hospital that this patient's positive TST is secondary to a vaccination at birth was incorrect. Positive PPD in this patient should have prompted further investigations.

## 2. Background

It is interesting to note that Thomas Addison was in fact seeking an anatomic basis for pernicious anemia rather than the biochemical effects of adrenal insufficiency when he published his seminal paper on the subject. The eleven patients he described in his report all had tuberculosis of the adrenal glands [3]. This consumption has since receded to the background of ailments that afflict the Western world and today is generally considered a disease of immigrants from endemic areas, the immunocompromised or the destitute. In the developing world, however, tuberculosis continues to account for about 20–30% of cases of Addison's disease [4].

The clinical presentation of primary adrenal insufficiency is protean, and an underlying infectious etiology can further obscure the manifestations. The most frequent manifestations are weakness, fatigue, anorexia, weight loss, nausea, vomiting, hypotension, and skin hyperpigmentation (present in 60–100% of patients) [5, 6]. Understandably any of the above symptoms or signs could be easily missed or attributed to the primary infectious process itself.

In the developed world, about 10% of cases of Addison's disease have an infectious etiology; however there are few data available regarding the frequency of organisms that cause clinical adrenal insufficiency. HIV/AIDS and opportunistic infections like cytomegalovirus are the most commonly cited causes following tuberculosis. Various fungi like Cryptococcus, Histoplasma, Coccidioides, Paracoccidioides are also described to involve the adrenal glands in several case reports (Table 1).

## 3. Tuberculosis

*Mycobacterium tuberculosis* complex spreads to the adrenal glands hematogenously. Clinical manifestations may take years to become apparent, and asymptomatic infection is not uncommon. Adrenal involvement was found in 6% of patients with active tuberculosis in an autopsy series [7]. More than 90% of the gland must be destroyed before insufficiency appears [8]. The widespread use of computed tomography has improved our understanding of the patterns of involvement of the adrenal gland in tuberculosis. The majority of patients with active or recently acquired disease (<2 years) have bilateral adrenal enlargement, while calcification and atrophy are the norm with more remote infection or inactive disease [8, 9].

That the adrenals can be enlarged in patients with pulmonary tuberculosis without active involvement of the glands has been demonstrated in various studies. Stress and inflammation could be potential reasons. The activity of the hypothalamic pituitary axis (HPA) has been the subject of numerous studies. The lack of a uniform definition of a “normal cortisol response” to ACTH stimulation has perhaps contributed to some of the heterogeneity in the results. Keleştimur et al. studied the HPA axis in 27 patients with active pulmonary tuberculosis. They also compared responses to 1 mcg and 250 mcg ACTH stimulation. Cortisol responses were consistently higher in the cases when compared to controls [10]. A more recent study by Laway et al. found significantly lower basal and stimulated cortisol levels in active pulmonary tuberculosis when compared to controls, as well as enlarged adrenal glands. None of the patients had clinical adrenal insufficiency. Both of these findings improved after successful antituberculous treatment [4]. However, the absence of serum albumin levels and the lack of adrenal biopsies in the cases limit the interpretation of the results. Other studies have also demonstrated a reduction in adrenal gland size after successful treatment of pulmonary tuberculosis [11].

When tuberculosis results in overt adrenal insufficiency, antituberculous chemotherapy does not appear to restore function. One must also be cognizant of the effect of rifampin, a potent hepatic enzyme inducer on the metabolism of glucocorticoids. Failure to increase the dose of steroid replacement therapy may result in the development of adrenal crisis [1]. Adrenal biopsy is not necessary for primary adrenal insufficiency with bilateral adrenal enlargement in a patient with proven extra-adrenal tuberculosis. However, about 12% of patients with adrenal tuberculosis have no evidence of active extra-adrenal tuberculosis [12]. Adrenal biopsy is generally necessary in these patients to prove adrenal involvement by tuberculosis.

## 4. HIV Infection

HIV infection affects the adrenal gland in multiple ways. Apart from direct infection, opportunistic infections and antiretroviral medications also have a significant effect on the adrenal glands.

Adrenal insufficiency is prevalent in 17% of patients admitted with AIDS [13]. Due to its high prevalence, recommendations have been made to screen for adrenal insufficiency in HIV patients with symptoms [14]. Most common causes of adrenal insufficiency are infections like CMV,* Mycobacterium tuberculosis* and MAI,* Cryptococcus neoformans*,* Histoplasma capsulatum*,* Pneumocystis jirovecii*, and* Toxoplasma gondii*, neoplastic diseases (Kaposi's sarcoma and lymphoma), and bilateral adrenal hemorrhage [15, 16]. Few drugs used for the treatment of HIV infection (protease inhibitors) and drugs used to treat opportunistic infections like rifampicin, ketoconazole, and cotrimoxazole may exacerbate manifestations of primary adrenal insufficiency [17, 18]. Studies have shown decreased level of cortisol, adrenal androgens, and mineralocorticoids in patients infected with HIV [19].

HIV infection can also lead to secondary adrenal insufficiency in advanced stages of the disease by decreasing pituitary and adrenal responses to CRH [20]. Opportunistic infections like CMV,* Mycobacterium tuberculosis*,* Toxoplasma gondii*,* Cryptococcus neoformans*, and* Pneumocystis jirovecii* can also infiltrate the pituitary leading to multiple endocrinopathies.

Treatment of adrenal insufficiency in HIV infection includes hydrocortisone and fludrocortisone (if there is evidence of mineralocorticoid insufficiency).

There are cases of Cushing's like phenotype in patients treated with antiretroviral drugs (protease inhibitors and NNRTIs) often referred to as “pseudo-Cushing's” [21]. A normal cortisol response to the dexamethasone suppression test differentiates pseudo-Cushing's from Cushing's syndrome. Studies have also shown elevated levels of basal plasma cortisol in untreated HIV patients when compared to healthy individuals. The postulated mechanisms include stress due to HIV infection, increased cytokines resulting in stimulation of HPA axis, and reduction in cortisol catabolism [22].

Adrenal tumors found almost exclusively in HIV patients include Kaposi's sarcoma which is secondary to coinfection with the oncogenic human herpes virus type 8 (HHV8) and non-Hodgkin's lymphoma (high-grade malignant B phenotype) which could be secondary to Epstein-Barr virus (EBV) [23, 24].

## 5. Human Cytomegalovirus Infection

Human cytomegalovirus (HCMV) has been frequently identified as a cause of adrenal insufficiency, especially in patients with HIV/AIDS. The virus has been shown by Trevisan et al. to infect normal human adrenocortical cells and induce cytopathic changes [25]. It also acts as an inducer of steroidogenesis which may explain the discordance between the high rates of CMV adrenalitis in immune suppressed patients in autopsy studies and the relatively rare diagnosis of adrenal insufficiency ante mortem. The virus causes the greatest damage at the cortex-medulla junction [26]. While adrenalitis may be the sole manifestation, the disease is usually disseminated. There have been case reports of exacerbation of CMV infection in patients of adrenal insufficiency after starting glucocorticoids most likely due to the immunosuppressive effect [27, 28].

## 6. *Histoplasma capsulatum*

Adrenal involvement is typically seen with disseminated chronic histoplasmosis. In one report, primary adrenal insufficiency occurred in 41.3% of adrenal histoplasmosis cases [29]. Histoplasmosis often coexists with HIV-AIDS and is more commonly seen in the immunocompromised, posttransplant and elderly populations [30]. Histoplasmosis presents with similar constellation of clinical features as tuberculosis and is often missed. Furthermore, it also shares pathological characteristics of necrotizing granulomas and caseous necrosis with tuberculosis. Adrenal glands have bilaterally enlarged radiographic appearance and CT guided biopsy often confirms the diagnosis of adrenal histoplasmosis [31, 32]. In addition to tuberculosis, it can also be often mistaken for a lymphoma, underlining the importance of biopsy in these cases. Management includes treatment with amphotericin B followed by itraconazole (for disseminated disease) and replacement of glucocorticoid and mineralocorticoid if there is evidence of adrenal insufficiency.

## 7. Paracoccidioidomycosis

Paracoccidioides, a thermal dimorphic fungus, causes infection through the inhalation of infectious conidia. It is endemic in several South American countries. Two species,* P. braziliensis* and* P. lutzi,* are pathogenic in humans. While the acute form usually appears as progressive lymphadenopathy, the chronic form affects the skin, lungs, mucous membranes, and the adrenal glands [33]. Adrenal insufficiency occurs in a large number of patients (2.9%–48.2%) and has even been reported as the initial presenting feature [34, 35]. Autopsy series have demonstrated adrenal involvement in 85–90% of cases [35]. Clinical manifestations range from the asymptomatic to frank Addisonian crisis. This correlates with the extent of granulomatous involvement of the adrenal glands. Similar to tuberculosis, adrenal insufficiency typically persists even after treatment of the infection [35].

## 8. Other Causes of Adrenal Infections

### 8.1. Viruses

Many of the herpes viruses infect the adrenal gland including herpes simplex virus types 1 and 2, Epstein-Barr virus, and HCMV. This occurs usually in the setting of disseminated disease and may appear as adrenalitis. Disseminated HSV type 1 and 2 infections in neonates can be fulminant [36]. Murine models suggest the possibility of the adrenals being the initial seat of multiplication of these viruses [37].

EBV, HCMV, and Polyoma BK virus have been identified in resected adrenocortical tumors; the former has also been associated with lymphoma of the adrenal gland [38, 39].

Other commonly occurring viruses that can infect the adrenal glands include echoviruses which can lead to adrenal hemorrhage and necrosis in neonates [40].

The hemorrhagic fever viruses, although rare, can cause devastating damage to the adrenal glands. The Ebola virus, a filovirus, has been shown to cause liquefaction of the adrenals [41]. Other filoviruses and arenaviruses can also damage the adrenals by direct infection [42].

### 8.2. Fungi

Adrenal cryptococcosis occurs in disseminated cryptococcal infection, usually in the immunocompromised; however, there are case reports of adrenal cryptococcosis in healthy individuals as well. Pneumonia and meningitis are the most common presentations [43]. Like any other fungal infection, the adrenal glands are enlarged on CT scan and the diagnosis is confirmed by a CT guided biopsy [44]. Cryptococcal antigen titers are invariably high and can be used as a biomarker for disease resolution on follow-up [45]. A 6-month course of fluconazole appears to be effective [46]. In contrast to tuberculosis and histoplasmosis, adrenal insufficiency is often improved with resolution of the disease. Adrenal cryptococcal infection resistant to antifungal therapy may respond to adrenalectomy [47].

*Pneumocystis jirovecii* is an infrequent cause of adrenal insufficiency even in patients with defective cell mediated immunity such as patients with HIV/AIDS. However, it has been known to cause fatal adrenal crisis in the apparently immunocompetent host as well [48].

*Blastomyces dermatitidis* is a thermal dimorphic fungus that is the North American counterpart of Paracoccidioides in terms of its pathogenesis and propensity for establishing chronic systemic infection. Although pulmonary infection is the most common presentation, it frequently affects the skin, bones, adrenal glands, and the genitourinary system. Almost any organ system may be involved. It appears that subclinical infection of the adrenal glands is more common than overt insufficiency [49]. About 10% of cases in autopsy series revealed adrenal gland infection [50]. Amphotericin B and itraconazole are the drugs of choice in disseminated disease.

### 8.3. Bacteria

Atypical mycobacteria have been isolated from adrenal glands in patients with HIV/AIDS, however, given the multiple etiologies for adrenal insufficiency and frequent coinfection with other organisms that are known to cause destruction of the adrenal glands; however, it is difficult to establish a causal relationship [15].

The Waterhouse-Friedrichsen syndrome deserves special mention. It is a form of acute adrenal insufficiency that occurs in the setting of bacterial sepsis resulting in adrenal hemorrhage. A number of bacteria are associated with this entity including* N. meningitidis*,* H. influenza*, pneumococcus,* P. multocida*,* K. oxytoca*,* S. aureus*,* Capnocytophaga canimorsus*, Ewingella, and group A streptococcal infections. The organisms are rarely isolated from the adrenal glands at autopsy [51–54].

### 8.4. Parasites

Of all the organisms mentioned in this review, parasites are perhaps the least commonly reported causes of adrenal infection in the United States. Microsporidia have been reported to cause necrotic lesions in the adrenal glands, particularly in patients with AIDS [55]. Echinococcosis (hydatid disease) is responsible for about 7% of all adrenal cysts. Treatment is surgical excision followed by several months of chemotherapy with oral albendazole to prevent recurrence [56, 57]. Visceral leishmaniasis is another cause of cystic adrenal disease.* Trypanosoma cruzi*, the causative agent of Chagas' disease, has been shown to infect the adrenal gland. Studies have postulated that the adrenals may serve as a reservoir for* T. cruzi* and parasitemia in the central adrenal vein has been correlated with the development of chronic chagasic cardiomyopathy [58, 59]. African trypanosomiasis has been associated with polyendocrinopathies including hypogonadism, hypothyroidism, and adrenal insufficiency. This can result from a primary glandular or secondary (central) involvement. In one case series of 137 Ugandan patients, treatment of the infection resulted in recovery of adrenal and thyroid function, but hypogonadism tended to persist for years [60].

## 9. Conclusion

The adrenal glands can be affected by a wide range of organisms through multiple mechanisms including direct infection as well as disturbance of the HPA axis due to physiologic stress and cytokine release being the most common. Many of the diseases described in this review appear as chronic illnesses that have manifestations similar to adrenal insufficiency; therefore, very high index of suspicion is required for making the diagnosis of adrenal insufficiency in these patients. Bilateral adrenal enlargement is a feature of a number of these illnesses, especially the granulomatous infections. It is interesting to note that bilateral adrenal enlargement does not necessarily indicate adrenal infection but may be merely reflective of the response to stress. Successful treatment often results in the reduction of gland size. Once adrenal insufficiency has set in, however, the adrenal hypofunction tends to persist despite appropriate treatment of the underlying infection.

The biochemical diagnosis of adrenal insufficiency is often complicated by acute illness, which ironically is often the setting for it. Lack of uniform definitions and availability of reliability of assays used to measure ACTH and cortisol levels were the limitations encountered in a number of studies. Current guidelines recommend using a 250 mcg postintravenous corticotropin stimulated cortisol level of >18 mcg/dL as a cut-off to rule out primary adrenal insufficiency. In the setting of critical illness a serum cortisol level less than 25 mcg/dL or an increment of less than 9 mcg/dL 30 minutes after a 1 mcg intravenous corticotropin injection may be suggestive of adrenal insufficiency [61].

Steroid replacement strategies are the same irrespective of the etiology, but special attention must be paid to patients on rifampin, isoniazid, azole antifungal agents, and certain antiretroviral agents (ritonavir). These agents significantly impact steroid catabolism by interfering with the cytochrome P 450 system [62].

“The leading and characteristic features which merit attention are anemia, general languor and debility, remarkable feebleness of the heart's action, irritability of the stomach and a peculiar change of the color in the skin, occurring in connection with a diseased condition of the suprarenal capsules.” Dr. Addison's classic description can scarcely be improved upon today. It cannot be emphasized enough that the diagnosis of adrenal insufficiency is a clinical challenge and requires a high index of suspicion, especially in the setting of an infectious process [3].

## Figures and Tables

**Table 1:** Salient features of various adrenal infections.

Organism	Imaging findings	Comments
*Mycobacterium tuberculosis *	Bilateral adrenal enlargement (active infection). Atrophy and calcification in remote infection	Adrenal enlargement improves with treatment; adrenal insufficiency does not. Steroid dose should be increased if on rifampin

HIV	Depends on the etiology (multiple OIs can involve the adrenals)	Adrenal insufficiency due to viral, fungal, mycobacterial infiltration. “Pseudo-Cushing's” due to antiretroviral drugs and impaired cortisol metabolism

*Histoplasma capsulatum *	Bilateral adrenal enlargement	Nearly 50% have adrenal involvement

*Paracoccidioides *	Bilateral adrenal enlargement	Endemic in South America. Adrenal insufficiency does not always improve with treatment of the infection

*Blastomyces dermatitidis *	Bilateral adrenal enlargement	Similar to paracoccidioidomycosis, overt adrenal insufficiency is less common

Human cytomegalovirus infection	Variable	One of the most common adrenal infections in patients with AIDS. Insufficiency can manifest even when the patient is on glucocorticoid replacement

Bacterial sepsis	Adrenal hemorrhage	A number of bacteria are associated with the Waterhouse-Friderichsen syndrome. Most commonly seen when encapsulated organisms cause overwhelming sepsis

*Echinococcus* sp.	Adrenal cysts	Causes 6-7% of all adrenal cysts. Treatment is with surgery and albendazole

*Trypanosoma* sp.	Variable	Adrenals may be the reservoir for *T.cruzi* while *T.brucei* (African sleeping sickness) causes mixed central/peripheral adrenal insufficiency
